# Accidental Cut-Throat Injury by a Broken Glass: Bloodstain Pattern Analyses and Autopsy Findings

**DOI:** 10.7759/cureus.51945

**Published:** 2024-01-09

**Authors:** Biliana Mileva, Metodi Goshev, Mihaela Georgieva, Ivan I Tsranchev, Alexandar Alexandrov

**Affiliations:** 1 Department of Forensic Medicine and Deontology, Medical University Sofia, Sofia, BGR; 2 Department of Forensic Medicine and Deontology, Medical University of Plovdiv, Plovdiv, BGR

**Keywords:** accidental cut-throat injury, forensic autopsy, neck zones, bloodstain pattern, crime scene investigation, death scene investigation, neck injury, neck vascular injury, cut-throat injury

## Abstract

Injuries in the neck region are rarely observed in forensic practice, especially of accidental origin. Primarily, such cases are associated with homicide or suicide. The neck region comprises different and vital anatomical structures, and even minor trauma could be lethal. In the absence of witnesses to the accident, each finding is of utmost importance, from the death/crime scene investigation - bloodstain patterns and trace evidence - to careful examination of the deceased body. The forensic pathologist has the challenging task of analyzing all the findings to make a statement concerning the cause and manner of death and, if there is something suspicious about the current case, to inform the relevant authorities.

## Introduction

Penetrating neck injuries are relatively rare compared to injuries to other parts of the human body. Globally, those types of injuries account for approximately 5% to 10% of all traumatic injuries [1,2]. In vertebral anatomy, the throat is the anterior part of the neck, in front of the vertebral column. It comprises the larynx, trachea, pharynx, esophagus, cricoid, thyroid, hyoid, and vital blood vessels - the carotid artery and jugular vein [3,4]. Cut-throat injuries are inflicted by objects with sharp tips and sharp edges, represented by different objects such as razors, knives, broken bottles, or glasses [5,6]. Since the neck region has many anatomical structures, the neck is divided into three zones - I, II, and III for descriptive and clinical management purposes [1,5]. Zone I is situated from the level of the cricoid cartilage to the clavicles. Zone II injuries occur in the area between the cricoid and the mandible's angle, with the latter being the most easily accessible area, thus making it the most commonly injured region of the neck. Zone III injuries are between the mandible's angle and the base of the skull [1,2,5].

Regardless of the region affected, penetrating neck trauma is always a potentially lethal one. In such cases, death occurs due to profuse bleeding, air embolism, or due to inhalation of effused blood [3,6]. Cut-throat injuries may be intentional (homicidal or suicidal) or accidental [2,3,7-9]. Accidental cut-throat injuries are infrequent and are mostly related to a road traffic accident or fall from a height [2,10].

## Case presentation

Case

A young girl was found dead by her mother in the corridor of their house. It is highly suspected that the accident happened when the girl was running/playing around the kitchen table and jumping over the chairs, which was her usual activity. During this, the girl accidentally broke a glass into pieces. She lost balance, fell over the broken cup, and accidentally cut her throat. The mother heard the noise in the kitchen and immediately ran to see what was happening, but it was too late. The girl was found unresponsive.

Crime scene investigation

The Kitchen

The kitchen table was situated right in front of and perpendicularly to the kitchen door. There were a few small fragments of the broken glass on the table. The tablecloth at the corner of the table was soaked with blood with a few distinguishable satellite spatters. On the floor, there were several glass fragments stained with blood, one more significant in size and irregular in shape. Next to it was sizeable bloody staining on the carpet and the parquet, with a distinguishable wavelike pattern at the side next to the glass fragment. The other side of the stain was without any specific pattern but with multiple, circular drops of blood, satellite spatters, and spines on the periphery of the stain and the nearly situated wall. At the end of the kitchen chair, there was minor staining with blood and a few circularly shaped blood drops on the wooden part. Small bloody steps were noticed over the carpet, starting from the bloodstained carpet, passing by the kitchen table, and leading to the corridor, with a few spherical blood drops on the left side.

The Corridor

The bloody steps became paler in the corridor. At the end of it, in front of the door, was situated the body of the little girl - parallel to the door, with the head pointing opposite to the door handle. There were pale blood stains on the door, with a “trail” appearance with few satellite spatters. The blood was flowing down obliquely, starting from the door handle. Around the head and neck of the girl, there was a pool of blood.

Autopsy findings

The girl was wearing her pajamas; the upper part of it was abundantly soaked in blood, and then there were a few elongated, elliptical blood drops on the left trouser leg and a few, circular blood drops on the upper side of her left sock. The lower part of both socks (the foot) was completely soaked in blood. The clothes were carefully removed, packed, and sent to the police, according to standard practice. The lividities were scanty, represented by small patches, purplish in color, situated on the back and posterior parts of the thighs, and disappearing upon application of pressure. The rigor mortis was well marked. The body was cold.

**Figure 1:**
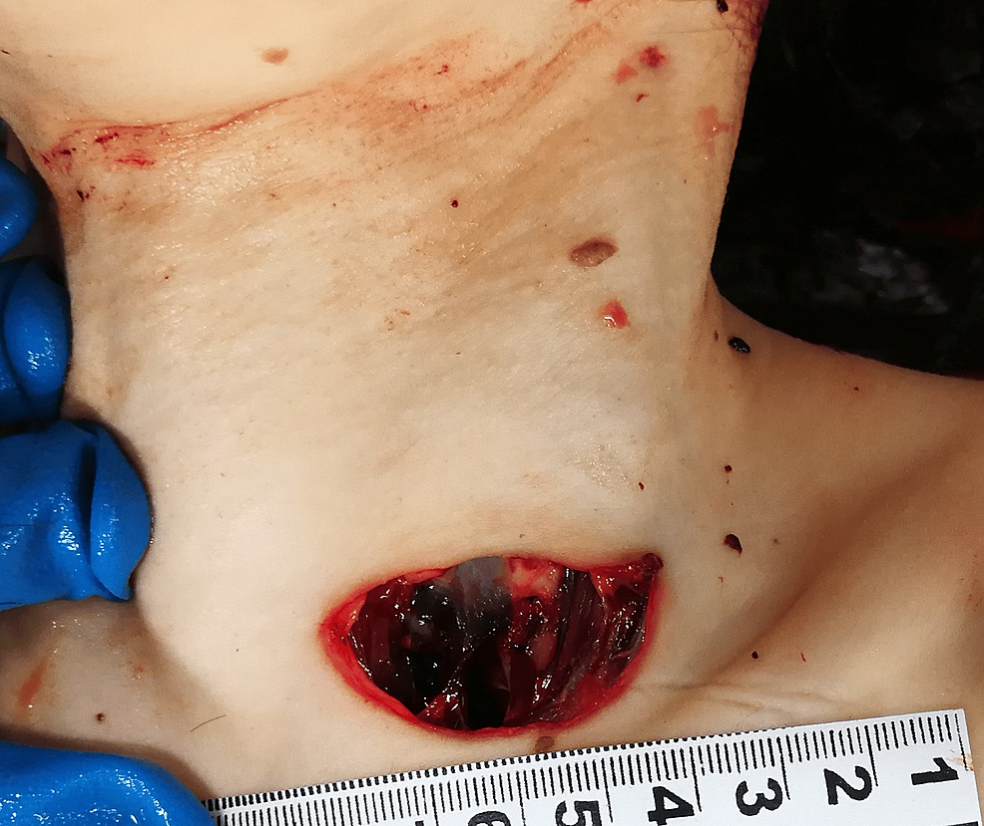
Injury (gapping wound) to the anterior aspect of the neck

On the anterior aspect of the neck, in zone I on the left, below the cricoid cartilage, there was a gaping wound situated relatively perpendicular to the body's midline. After approximating the wound margins, the wound was measured to have a length of about 4.2 cm. The margins were smooth and relatively clean cut but with isolated serrations and abrasions in some places, mainly on the lower margin. The left (medial) angle was rather sharp, and the other was slightly round. The wound was of different depth, evidently more superficial in its lateral (right) part and deeper in its medial end. No tissue bridges were present. There was another small puncture wound with a diameter of about 0.2 cm, situated at a distance of about 122 cm from the feet, right below the angle of the lower jaw (Figure 1).

**Figure 2:**
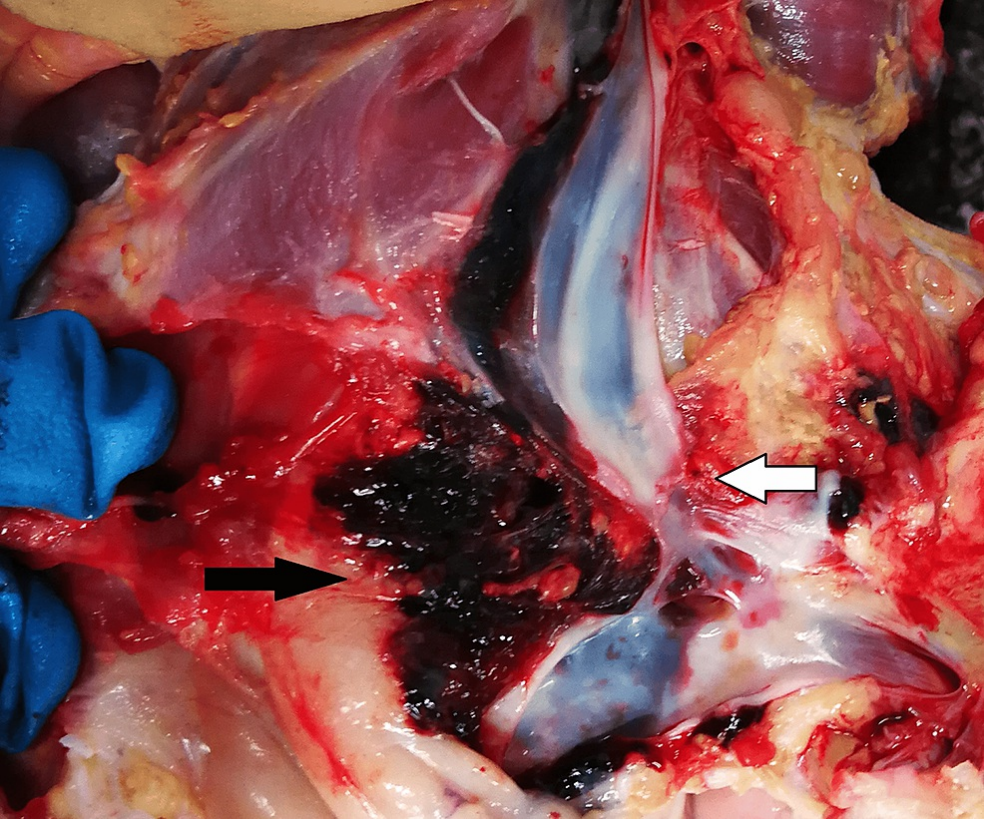
The cut internal jugular vein (white arrow) and the bruise of the soft tissues (black arrow)

**Figure 3:**
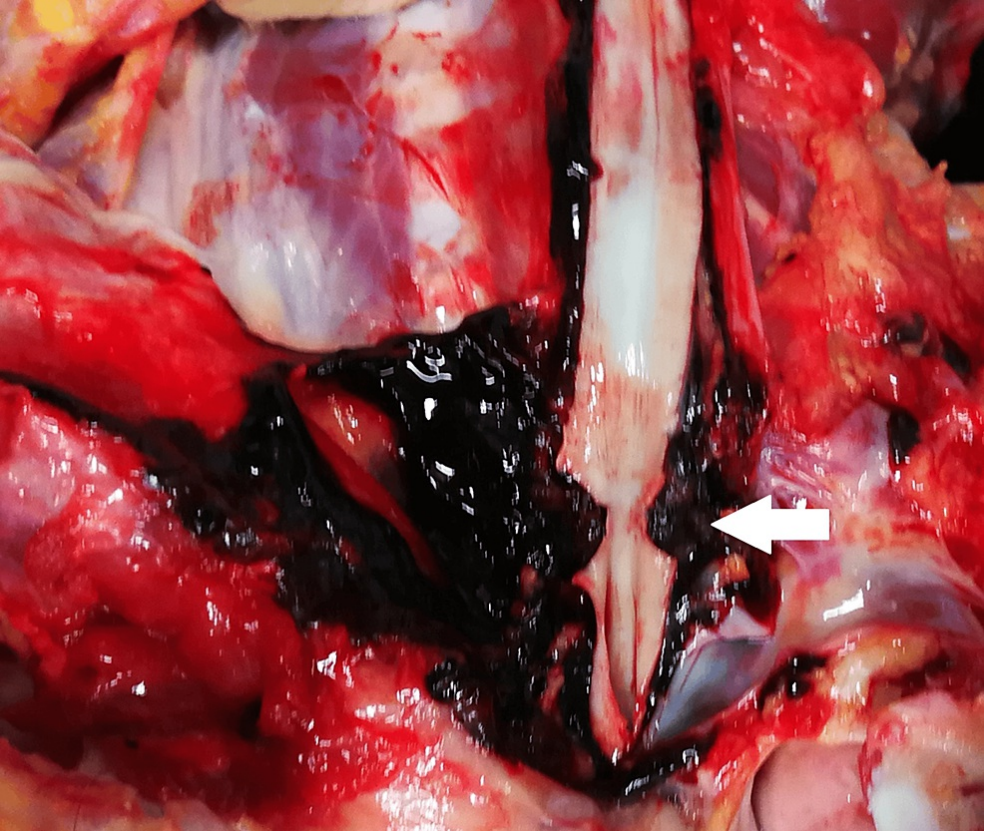
Cutted arteria carotis communis

No other traumatic injuries were noted during the external examination of the body. The soft tissues of the neck were carefully removed layer by layer to examine the affected anatomical region and structures. The soft tissues below and around the wound were intensively bruised, and the sternocleidomastoid muscle had been cut in its lower part. The internal jugular vein and the arteria carotis communis had been almost completely cut (Figure 2 and Figure 3).

During the internal examination of the body, signs of severe blood loss were noticed - pale and dry internal organs. The test for air embolism and the toxicology analyses were negative. DNA analyses confirmed that the blood found at the death scene was the girl's blood.

## Discussion

Several studies presented in the literature provide information about cut-throat injuries. They are described in Table 1.

**Table 1:** The manner of death and the number of dead people according to different studies

Authors	Manner of death		
	Suicide	Homicide	Accident
Rao Dinesh [3]	2	72	-
Kundu RK et al. [11]	40	13	7
Bhattacharjee N [12]	11	11	4
Santhaiah K [13]	8	6	1
Aich M [5]	7	48	12
Gilyoma JM [2]	34	54	10
Ahmed MA [9]	12	24	12
Beigh Z [14]	15	10	1

Bloodstain pattern analysis examines shapes and the categorization and distribution of bloodstain patterns. These analyses can give valuable information, help reconstruct the events of the crime/accident, and help evaluate the statements of witnesses and crime participants [15]. In the presented case, based on the fact that there were multiple fragments from the glass over the kitchen table, we conclude that the glass shattered over the table and not on the floor. Another finding was blood on the corner of the tablecloth with a few satellite spatters. This led us to the conclusion that the girl lost her balance and fell over the corner of the table, where the larger irregular in shape fragment injured her throat at that moment. Following this, the girl fell onto the floor, as well as the broken fragment of glass. The evidence in support of this theory is the large amount of blood over the floor with the typical appearance of arterial spurt - presents as a zigzag-like pattern next to the broken fragment of the glass - with the blood being accelerated by arterial pressure. The pattern reflects the course of the blood curve between the systolic (the arterial pressure first propels blood upward) and the diastolic phase (the decrease in pressure allows the blood to drop downward) [15,16].

**Figure 4:**
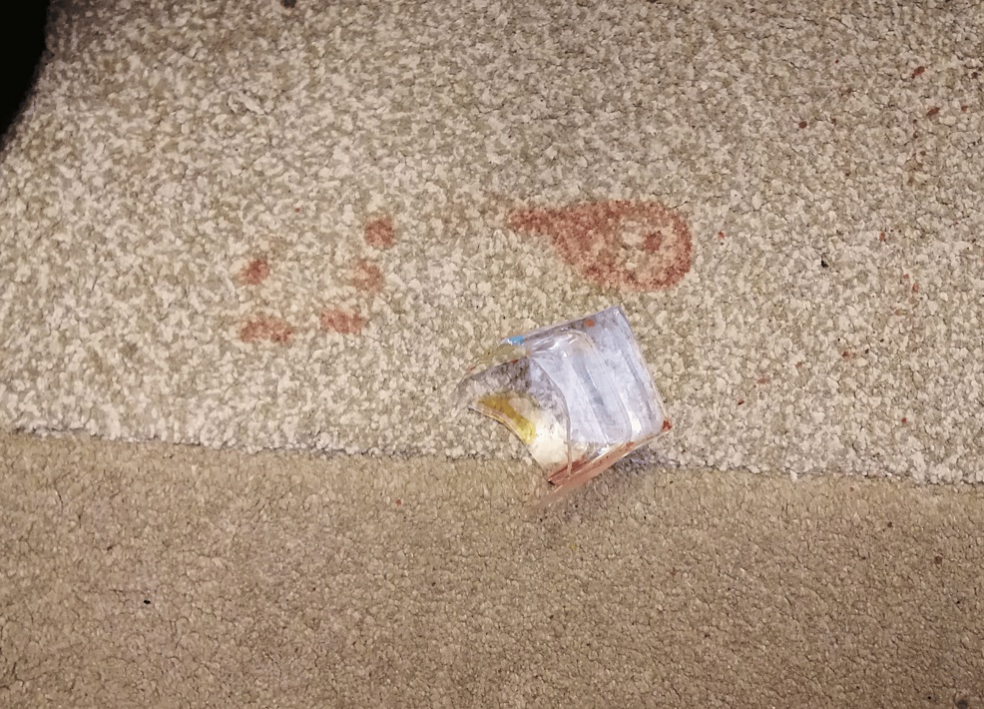
Bloody steps on the kitchen floor

Despite the profuse blood loss, the girl stood up, probably by supporting herself on the kitchen chair. During this process, the girl stepped in the gathered amount of blood on the floor, which is supported by the fact that the lower surfaces of her socks were all soaked up with blood and by the presence of bloody steps on the kitchen carpet, with a decrease of their intensity on the way to the corridor (Figure 4).

**Figure 5:**
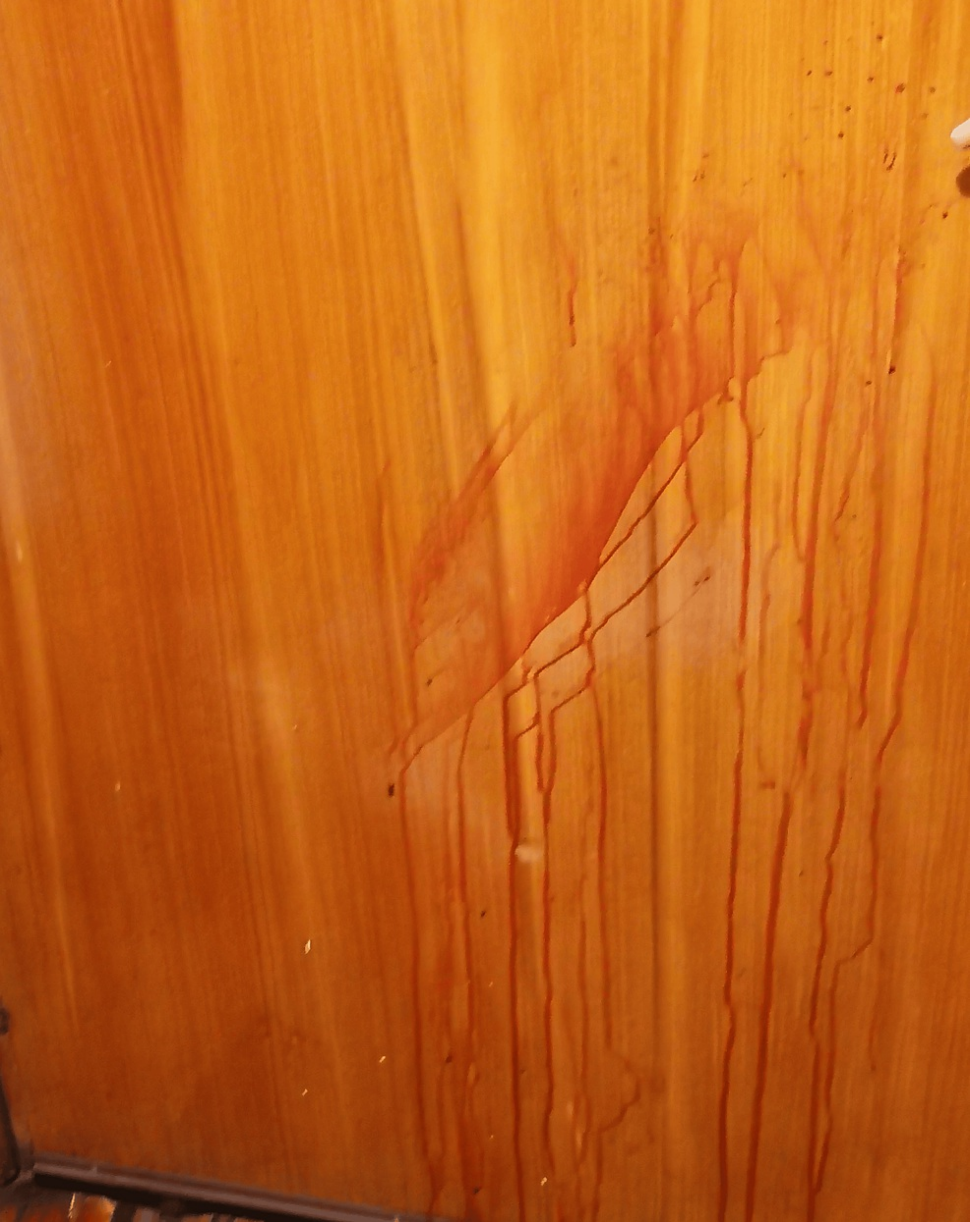
Door on the corridor – arterial spurt – “trail” appearance with some satellite spatters

Additional proof that the girl was standing for some time is the presence of drip stains over her clothes - mainly over her left trouser leg and the upper surface of her left sock. The drips were of different shapes, which can be explained by the different angles of falling - if the droplet falls at an angle of 90⁰, it will have an almost round shape; if it falls at an angle of less than 90⁰, it will more elliptic shape [15,16]. It is highly likely that on the way to the corridor, the girl was compressing her neck, trying to stop the bleeding. The girl reached the door of the corridor but had already lost a considerable amount of blood, lost consciousness, and died behind the door. The bloody stain on the door is typical for an arterial spurt but is not so intense due to the drop in blood pressure. The blood flows down due to gravity (Figure 5).

**Figure 6:**
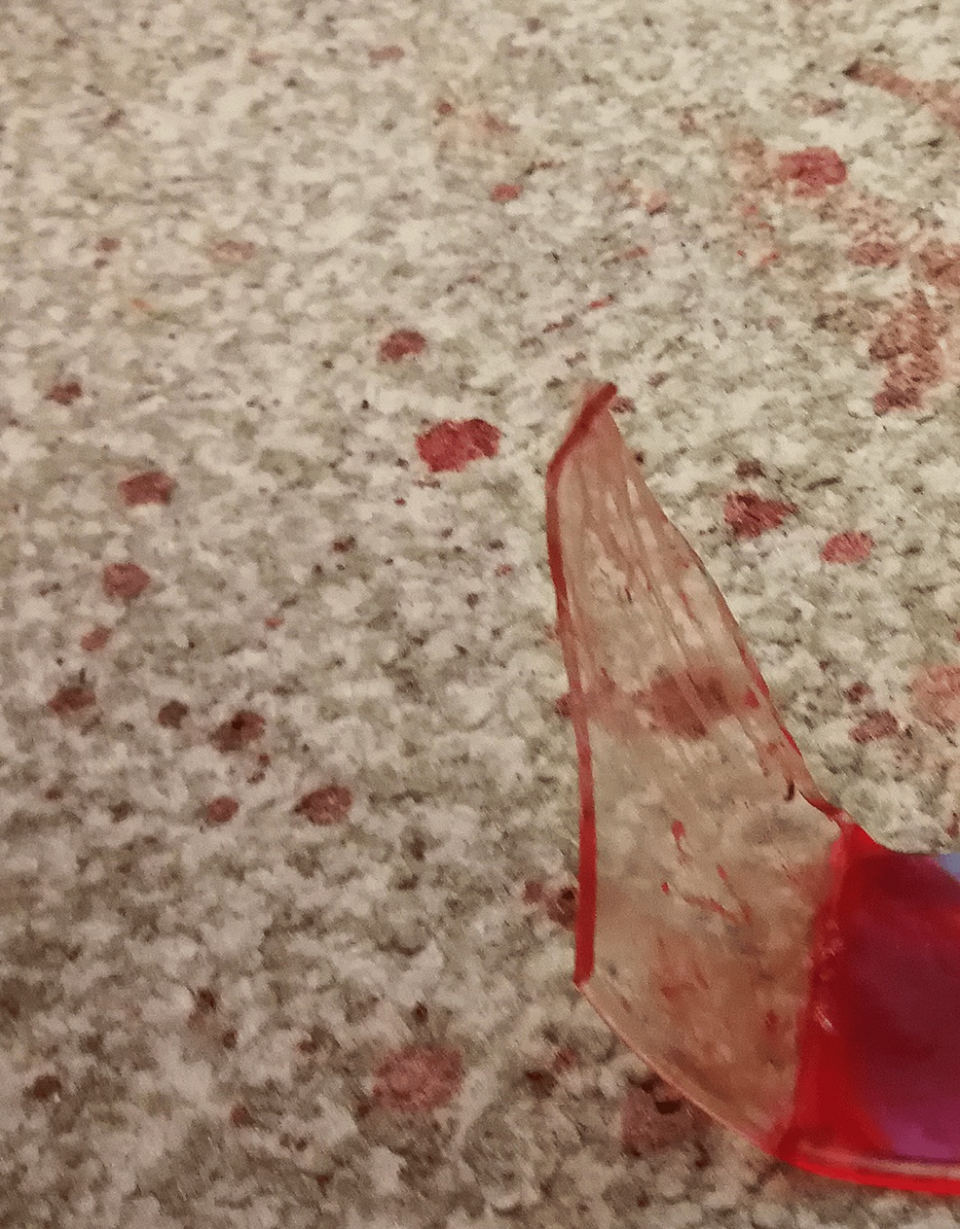
Fragment of the broken glass

No other stains were present at the death scene, which cannot be explained by the girl's actions or that support someone trying to alter the crime scene. The autopsy findings did not show other traumatic injuries over the girl's body supportive of a fight or struggle before her death, only the wound on the neck. The wound corresponded to being produced by the irregularly shaped broken glass found on the kitchen floor (Figure 6).

A smaller fragment of glass produced the other small puncture wound on the upper part of the neck. The cause of death was hemorrhagic shock due to the stab-incised injury of the neck. The prosecutor pronounced the manner of death to be accidental in origin.

Accidental cut-throat injuries are infrequent and are primarily associated with road traffic accidents and falls from heights, as previously mentioned. There are only a few reported cases in the literature similar to the one presented. Eugene Choi described the case of a 21-year-old girl who was found dead on the floor of her studio apartment in a pool of blood, naked, with a wound on her neck. The girl had fallen down the stairs onto the ground and injured her neck with broken glass [17]. Fracasso T et al. describe another peculiar death scene. A 31-year-old woman was found dead by her daughter, lying in the living room in a large pool of blood. During the autopsy, two incisions were present at the anterior aspect of the neck, corresponding to the sharp ends of a broken wineglass [18].

Cut-throat injuries, especially when there are no witnesses to the accident, raise suspicion of a possible homicide. The forensic pathologist has the challenging and vital task of carefully examining all the details from the death/crime scene to the autopsy of the corpse to establish the cause and manner of death and the mechanism of inflicted injuries [19,20].

## Conclusions

The presented case describes a rarely observed accidental injury in forensic practice - a cut-throat injury caused by a fragment of a broken glass. The study aims to raise awareness about the importance of bloodstain pattern analysis and interpretation of the autopsy findings in reconstructing the events.
